# Musical Sequence Learning and EEG Correlates of Audiomotor Processing

**DOI:** 10.1155/2015/638202

**Published:** 2015-10-07

**Authors:** Matt D. Schalles, Jaime A. Pineda

**Affiliations:** ^1^Department of Cognitive Science, University of California San Diego, La Jolla, CA 92093, USA; ^2^VA Northern California Health Care System, Martinez, CA 94553, USA; ^3^Department of Neuroscience, University of California San Diego, La Jolla, CA 92093, USA

## Abstract

Our motor and auditory systems are functionally connected during musical performance, and functional imaging suggests that the association is strong enough that passive music listening can engage the motor system. As predictive coding constrains movement sequence selections, could the motor system contribute to sequential processing of musical passages? If this is the case, then we hypothesized that the motor system should respond preferentially to passages of music that contain similar sequential information, even if other aspects of music, such as the absolute pitch, have been altered. We trained piano naive subjects with a learn-to play-by-ear paradigm, to play a simple melodic sequence over five days. After training, we recorded EEG of subjects listening to the song they learned to play, a transposed version of that song, and a control song with different notes and sequence from the learned song. Beta band power over sensorimotor scalp showed increased suppression for the learned song, a moderate level of suppression for the transposed song, and no suppression for the control song. As beta power is associated with attention and motor processing, we interpret this as support of the motor system's activity during covert perception of music one can play and similar musical sequences.

## 1. Introduction

The performance of music recruits and synchronizes many neural systems, integrating motor output through auditory, somatosensory, and oftentimes visual input. Listening to sounds can easily stimulate the motor system to act in the form of head nodding, foot tapping, and dancing. Functional imaging of silent piano performance and passive listening to piano song reveals shared recruitment of auditory and premotor cortices [1–3], for pianist and nonpianist alike. Activity in these cortical regions is also modulated in task dependent measures. Subjects engaged in tapping along with simple beats exhibited simultaneous increases of blood flow to auditory and dorsal premotor cortices when the complexity of tapping beat increased [4]. Zatorre [5] proposed extending the definition of the auditory system to include interactions with other cognitive systems, such as motor and multisensory networks, to account for the distributed nature of cortical responses during auditory and musical perception. A growing body of evidence suggests that neural representations of movements and sounds may become linked through mechanisms such as Hebbian learning or as an emergent property of an auditory-motor loop [6]. The audiomotor system is adaptable and exhibits associations of novel sound-action pairings on short [7] and intermediate [8] time scales. If the motor system is routinely recruited for musical or auditory processing, what might it contribute as part of a distributed auditory network? Or to put it another way, what aspects of musical experience might be explained by a distributed audiomotor system which are not explained by an auditory system without motor contributions?

The audiomotor system is sensitive to rhythmic aspects of music such as timing and metrical complexity [9, 10] and also melodic information, both in terms of timbre of instruments [11] and pitch of melodic notes associated with motor actions [8]. Tracking metrical and melodic information requires accumulation of auditory information over time, which is used to generate expectations of future notes at precise time intervals. Neural processes track musical progressions and respond to violations of melodic or harmonic expectations with similar latency as semantic violations in language [12]. This suggests that we might process music as a hierarchically organized sequence of information, similar to language and motor programs [13, 14].

In the present study we asked whether the audiomotor system might be sensitive to sequential ordering of information in a musical passage, such that it could help generate top-down predictions for incoming auditory stimuli. Previous hypotheses posit that the motor system might contribute top-down predictive information in the form of a metrical grid [10, 15] or predicting the occurrence of a rhythmic beat or pulse [6] in music. This model is supported by in vivo multiunit recordings in monkeys during a visuomotor task describing initial feedforward communication from sensory to frontal cortices, followed by sustained feedback from frontal to sensory cortices [16]. While visual and auditory systems have different functional and anatomical connections with the motor system, it may be reasonable to assume that this feedforward and feedback dynamic may be representative of a supramodal sensorimotor loop.

As the audiomotor system is sensitive to both rhythmic and melodic aspects of music, we focused our present work on the sequencing of melodic information for two primary reasons. The first is that previous imaging work [8] demonstrated simultaneous activation of superior temporal auditory and premotor cortices during passive listening to a song that subjects had recently learned to play. Using a similar learn-to-play-by-ear task, we hypothesized that we could observe audiomotor engagement during passive listening using EEG measures that are sensitive to visuomotor engagement. And second, a melodic sequence affords an ideal manipulation of pitch information, while controlling for relative sequential information via use of a musical transposition of key. We hypothesized that if the audiomotor system is sensitive to melodic sequential information in the form of relative pitch intervals, then shifting the key or absolute pitch of a melodic sequence would still contain enough information associated with a motor sequence to engage the audiomotor system during passive listening.

Lahav et al. [8] report that the premotor and inferior parietal cortices respond preferentially when listening to a melodic passage that a listener knows how to play on the piano and exhibit little to no response when listening to a song unrelated to the one they learned how to play. Additionally, if subjects listen to a novel song that is composed from the same note set as the melody they learned to play, they exhibit reduced activation of the premotor and inferior parietal regions relative to listening to the learned song. The similarity was explained as a pitch-motion matching system, such that a single key press elicits a single sound and hearing the sound could trigger the association with the single motor act. An alternative explanation could describe the difference between learned song and novel song with learned notes as a difference in sequential information leading to reduced activity in motor planning areas.

In the present study, we asked whether the motor system is sensitive to preservation of the sequential ordering of musical information, even when the pitch information is altered. We used the piano ear-learning task developed by Lahav et al. [8, 17] and tested pitch-recognition-production matching before and after training. For the posttraining audiomotor system engagement, our work builds on past reports by using EEG measures of motor and sensorimotor system engagement [18], which could corroborate fMRI findings, as both BOLD signal increases and mu and beta band suppression in EEG (described below) are thought to index increases in cortical activity. If EEG is a good measure for audiomotor processing over the course of a musical phrase, it could encourage future study building on models of responses to discrete notes [19].

There is a strong history of EEG measures of motor system engagement during movement and the observation of movement. Reports from the late 1940s through the late 1970s describe mu power (8–13 Hz) over sensorimotor cortex as decreasing from a resting state during both actions and observation of actions, as discussed in [20–22]. The mu rhythm is suppressed during movement observation [23], performance of an action [24], and observation of object directed actions [25]. The mu rhythm is also suppressed during motor imagery [26]. The mu rhythm itself shares frequency properties with other brain rhythms, such as occipital alpha, but its intrinsic activity is functionally distinct, and source estimates localize it to the bilateral sensorimotor cortex surrounding the central sulcus [27]. The mu rhythm may be a good index of audiomotor processing as its suppression relative to baseline is associated with movement sounds, and combined sight and sound of actions suppress mu greater than either sensory input alone [28]. Mu rhythms as well as beta rhythms (15–30 Hz) are suppressed prior to a sound action (such as tapping on a drum) and exhibit rebound enhancement immediately after that action, whether the action is performed, observed, or heard [29]. Mu and beta both show increased phase coherence between motor, somatosensory, and auditory cortices when subjects synchronize movements to rhythmic sounds [30]. Additionally, beta rhythms appear to entrain to rhythmic sounds [31, 32] in auditory cortices and also exhibit similar patterns of suppression and enhancement for both listening and tapping [30] over motor areas. The synthesis of these studies indicates a similar neural process that is active during movement and listening, which is observed over the sensorimotor cortex. Additionally, sounds that have no clear movement association exhibit higher amounts of mu desynchronization after watching a video that associates that sound with a clear movement [7]. The mu rhythm also desynchronizes when expert pianists read sheet music [33]. Reading music is an activity that associates visual input, with a particular action, and becomes reinforced with auditory feedback.

We predicted that the mu and beta rhythms recorded from sensorimotor scalp would be suppressed maximally when a subject listens to a melody s/he knows how to play and fail to suppress or even show enhancement when listening to a melody unrelated to the learned song. Additionally, we predicted that listening to a transposed version of the learned melody would also elicit suppression; however the response might be attenuated relative to the learned melody itself, as previous findings indicate motor sensitivity to absolute pitch information, which is altered in the transposition.

## 2. Material and Methods

### 2.1. Participants

16 undergraduate students (nine female, mean age 19.9 years, 15 right handed), from the University of California, San Diego, completed the experiment in exchange for a combination of monetary compensation and course credit. One additional subject (female, 22 years old, right handed) completed the training and behavioral experiments, but not the electrophysiological component, and her data are included in behavioral measures. Subjects' handedness was self-reported, and additionally subjects were screened for head trauma, the use of psychiatric medication, and piano experience. In previous use of this task, only nonmusicians were included, but we included subjects with prior musical experience (*n* = 8), as long as all were inexperienced with the piano keyboard. We surveyed the number and type of instruments played, years spent playing, and whether subjects were currently playing music. Results of this survey are available in Table 1. All subjects were able to detect pure tones ranging between 250 Hz and 8 kHz at 30 db in both their right and their left ears. Subjects signed consent for procedures that were approved by the UCSD Institutional Review Board.

### 2.2. Song Stimuli

The same training song as described in Lahav et al. [8, 17] was implemented for the current study. For all the songs, synthesized backing instruments, guitar, bass, and drums, were composed following the score provided in [17] using Sonar Cakewalk Music Studio v4. The songs were each eight measures long and had a duration of 24 seconds at 80 beats per minute. The melodic line for each song was voiced by synthesized piano. The melody for each song was 15 notes long and comprised of a set of five notes (F-G-A-Bb-C), one for each finger on the playing hand. The transposed melody preserved the relative intervals between the notes in the learned song but shifted them into a set of notes one tritone, or half-octave, higher (B-Db-Eb-E-Gb) that did not overlap with the learned song note set. The control song was comprised of the same note set as the transposed song. The notes in the control song were arranged in a different sequence from the trained and transposed melodies while preserving the same total length of note duration and total changes in pitch height over the 8 bars. Backing rhythm guitar and bass lines in the transposed and control songs were likewise transposed up a tritone from the learned song.

### 2.3. Pitch-Recognition-Production Task

Sounds for this and subsequent behavioral tasks described here were presented over ambient speakers (Logitech 2.1 stereo computer speakers) which subjects adjusted to a comfortable level. Subjects were seated in a sound attenuated Faraday cage, positioned approximately 1.5 meters away from the speakers, in the center of the stereo field. Before the first training session and after the last, subjects listened to a series of 30 notes, randomly selected from the 5-note set (F-G-A-Bb-C) of the training melody. After each note, subjects were asked to press the corresponding key on the keyboard. Auditory feedback from the keyboard was disabled to prevent subjects from self-correcting their key presses as they progressed. This test was conducted to measure if subjects created behaviorally significant associations between sounds and discrete motor acts based on the one-note-one-finger training paradigm. Stimuli presentation and response recording were performed with Max/MSP 4.5.

### 2.4. Musical Training

For five consecutive days subjects practiced playing the melody line on a MIDI piano controller. On the first day, subjects were shown which five keys corresponded to the five notes used in the melody. One finger on the right hand was assigned to each of the five keys. Subjects were minimally supervised while figuring out the melody line by ear with the assistance of a computerized training environment. The song was introduced incrementally, starting with the first two measures. The subject was allowed to listen to and play along with exemplar piano lead over the two measures as many times as s/he desired. When ready, the subject would play the melody line over the backing instruments minus the exemplar piano lead. If the correct sequence of notes was played within 1st/16th note of the correct time, the computer informed the subject that he/she could move on to the next two measures. After a subject completed an additional two measures in the same fashion, the next training step was to play all of the previously learned measures in sequence. Thus they would first practice measures one and two and then measures three and four and then play measures one through four, until they could play the complete eight-measure melody. A training session was finished for the day when the subject could play the entire song with no mistakes. Time to completion was recorded for each training session. The training environment was coded in Max/MSP 4.5. A training session also involved listening to the transposed and control melodies before and after working through the piano sequence to control for familiarity by presenting each of the three experimental songs in their entirety the same amount of time to each subject each day.

### 2.5. EEG Task

After completion of training and the pitch-recognition-production task, subjects completed an EEG session where they listened to six-second-long clips (two measures) from the three songs. A pair of probe tones followed each song clip and subjects were asked to respond if the two tones were present in the previous song clip. Ten clips were created from each song, totaling 30 trials across the three conditions. A resting period of two seconds preceded the onset of song stimuli. A moving baseline for mu ratio calculations was collected from this prestimulus window, across all three conditions. Thirty bins of two-second baselines equaled the same number of time points as ten bins of six-second-long stimuli per experimental condition. Stimuli were presented pseudorandomly by Neurobehavioral Systems Presentation v. 13 software. Nineteen channels of EEG and two of ocular EMG were recorded using a Neuroscan Synamps system, according to the 10–20 standards for electrode placement (F3, Fz, F4, F7, F8, Fp1, Fp2, C3, Cz, C4, P3, Pz, P4, T5, T6, O1, O2, T3, T4, VEOG). Recordings were referenced to a digitally linked pair of mastoid electrodes and grounded at Fpz. Recordings were online bandpass filtered between 0.3 and 100 Hz and amplified by a gain factor of 1000.

## 3. Analysis

### 3.1. Behavioral

Length of time to complete training was recorded each day, and a training slope variable was calculated by a linear fit of the difference between the first and the second days of training. A series of pairwise correlations were calculated for the time to complete training on the first day, the training slope, years of previous musical experience, and pitch-recognition-production scores. We also calculated the difference between pitch-recognition-production scores from the posttest and the pretest sessions and added this to the correlation matrix. Correlations and analysis of variance were computed with MATLAB v. 7.10.

### 3.2. EEG

#### 3.2.1. Preprocessing

Offline data were processed in EEGLAB [34]. Data were band-passed between 3 and 40 Hz using the default FIR filter called by EEGLAB v. 12.0.2.4b. Epochs centered around onset of song stimuli were extracted to include the two-second baseline window before sound onset and the six seconds of duration of song stimulus. Independent component analysis (ICA) was performed (infomax algorithm) on the scalp channels, resulting in 19 components. Artifactual components, such as those representing eyeblinks or other head muscles, were visually identified and removed if they met the following three criteria: (1) irregular occurrence throughout the session, (2) scalp location indicating facial muscles, and (3) presence of abnormal spectrogram, such as extremely high-power low frequencies (eyeblinks) or disproportionately large power from 20 to 30 Hz (muscle contamination). After artifacts were removed, EEG signals were remixed from source space back into channel space for further analysis.

#### 3.2.2. Frequency Measures

All experimental conditions and baseline epochs were converted to frequency spectra using a fast Fourier transform with 0.5 Hz resolution. Frequency bands were summed with a trapezoid function for mu (8–13 Hz) and beta (20–30 Hz). Given the novel implementation of this behavioral task with EEG measures, it seemed prudent to explore other frequency bands outside of the mu and beta rhythms, such as theta (4–8 Hz) and gamma (30–40). Frequency band suppression was calculated as the log ratio of condition divided by baseline. This baseline ratio accounted for normalizing the differences inherent in spectral power due to interpersonal differences in scalp condition. The log transformation has the effect of turning ratios smaller than 1 into negative numbers, representing suppression below baseline, whereas enhancement is represented by positive values. Pairwise comparisons, correlations, and one-way ANOVAs were calculated in MATLAB v. 7.10 and repeated measure ANOVAs were computed in SPSS v. 20.0.

## 4. Results

### 4.1. Behavioral

**Figure 1:**
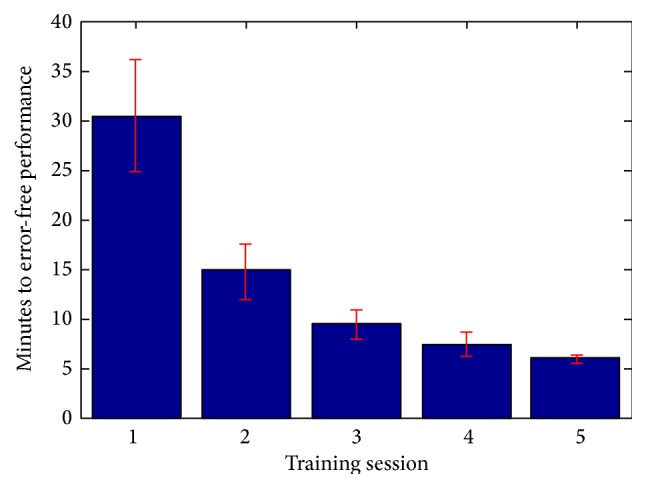
Mean amount of time to completion of training by day. Error bars (red) represent the standard error of the mean.

The amount of time it took subjects to learn the melodic sequence on the first day was highly variable (mean, 30.53 minutes; SD, 22.88). A significant effect of training day (*F*(4,80) = 12.02; *p* = 1.07*e* − 7) revealed a decrease in time to error-free performance and reduction in variability across all subjects over the five days of training (see Figure 1). Pearson correlation of length of time to error-free performance on the first day and years playing music shows a significant, negative slope (*r*(15) = −0.58, *p* = 0.01), but the relationship loses significance by the second day of training (*r*(15) = −0.39, *p* = 0.12). Previous musical experience was not correlated with pitch-recognition-production pretraining scores, posttraining scores, or the difference between them.

**Figure 2:**
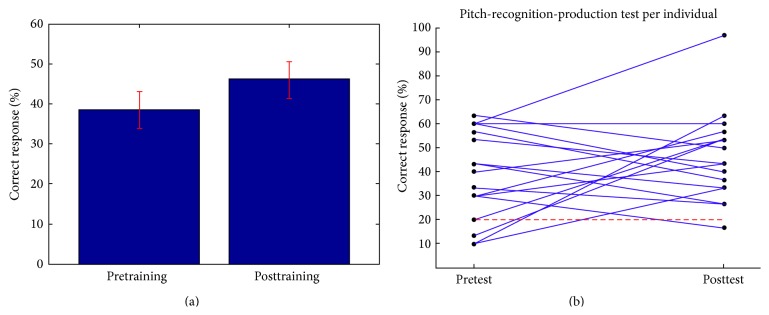
(a) Mean performance on the pitch-recognition-production test before and after musical sequence training. Chance performance is 20%. Error bars (red) represent the standard error of the mean (no significant difference between tests). (b) Individual performance with red dashed line denoting chance level performance.

The pitch-recognition-production matching test showed little improvement (see Figure 2) from the pretraining percent correct (mean, 38.63% correct; SD, 5.54) to the posttraining score (mean, 46.27% correct; SD, 5.51). The musically naive subject group showed a greater improvement in mean score, from 37.4 (SD = 5.9) percent correct to 48.5 (SD = 3.6) correct after training, compared with the musically experienced group who modestly improved from 40 (SD = 5.4) to 43.8 (SD = 7.3) percent. A mixed two-way ANOVA of within-subject factor of test (pre and post) and between-subject factor of musical experience corroborates this lack of significant difference between subject groups on the P-R-P test (*F*(1,15) = 0.38; *p* = 0.55).

### 4.2. EEG

Brain rhythm suppression was calculated at electrodes C3 and C4 following reports of mu activity at these recording sites [22, 35] and scalp projections of mu components are centered under these electrodes [36]. Repeated measures ANOVA with factors of electrode (C3 and C4), condition (control, learned, and transposed songs), and frequency (theta, mu, beta, and gamma) revealed a main effect of frequency (*F*(3,13) = 2988.37, *p* = 1.0*e* − 3) and a marginally significant interaction between frequency and electrode (*F*(3,13) = 2.65, *p* = 0.09). The theta and mu bands revealed consistent enhancement of power relative to baseline across all the conditions, whereas beta and gamma were generally suppressed relative to baseline. Across theta, beta, and gamma frequencies at these electrodes, the relative power was lowest for the learned song, followed next by control and then by transposed. Mu was the only frequency that exhibited higher power during the learned melody relative to the scrambled melody at C3. While we hypothesized the learned song would show greatest suppression, we did not expect the transposed song to elicit the least amount of suppression, even enhancement in some cases. Within-subjects comparisons revealed a significant main effect for frequency (*F*(3,45) = 6247.88, *p* = 1.0*e* − 4) and interaction between frequency and electrode (*F*(3,45) = 2.91, *p* = 0.05).

**Figure 3:**
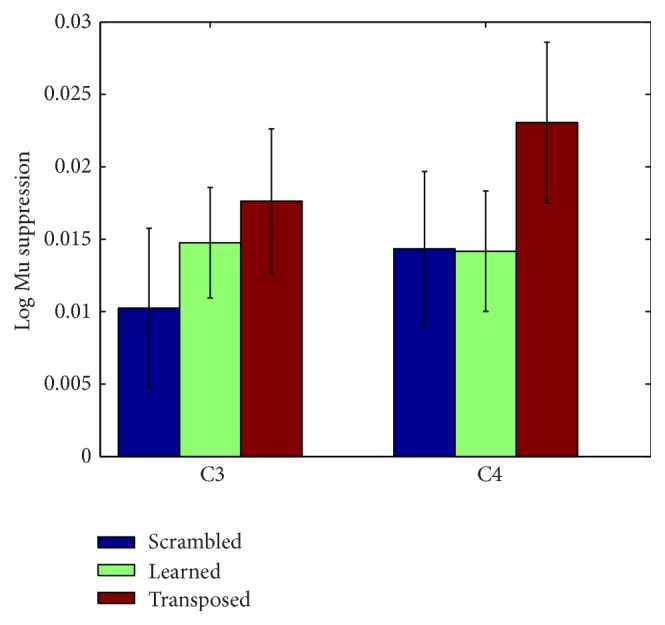
Mu suppression at electrodes C3 and C4. Error bars represent standard error of the mean.

**Figure 4:**
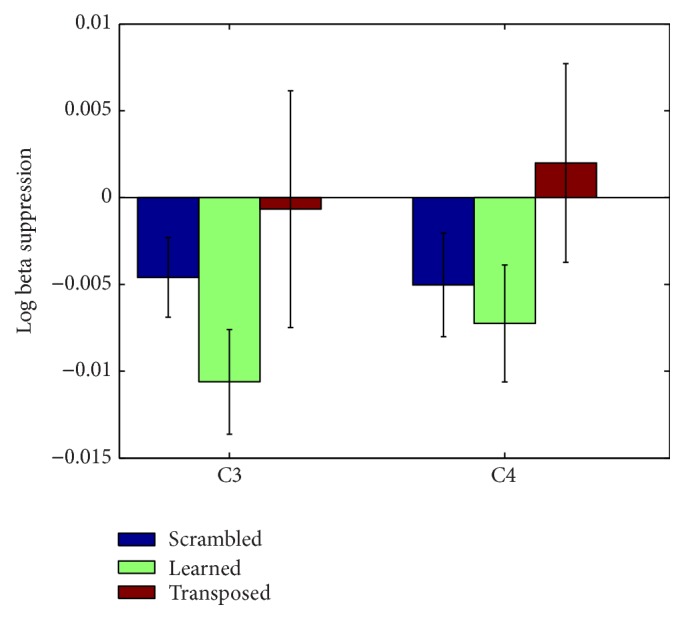
Beta suppression at electrodes C3 and C4. Error bars represent standard error of the mean.

The only frequency band that demonstrated suppression for all musical conditions was the beta band at electrode C3 (see Figure 4). Beta shows the same pattern of lowest log ratio power for the learned song, followed by control song and then transposed song. The pattern of relative power holds consistent for all three frequency bands at C3 and C4. The predicted results were not observed at electrode C3 or electrode C4 in the mu band (see Figure 3). Power values for each condition by frequency band and electrode are available in the Supplementary Material available online at http://dx.doi.org/10.1155/2015/638202.

**Figure 5:**
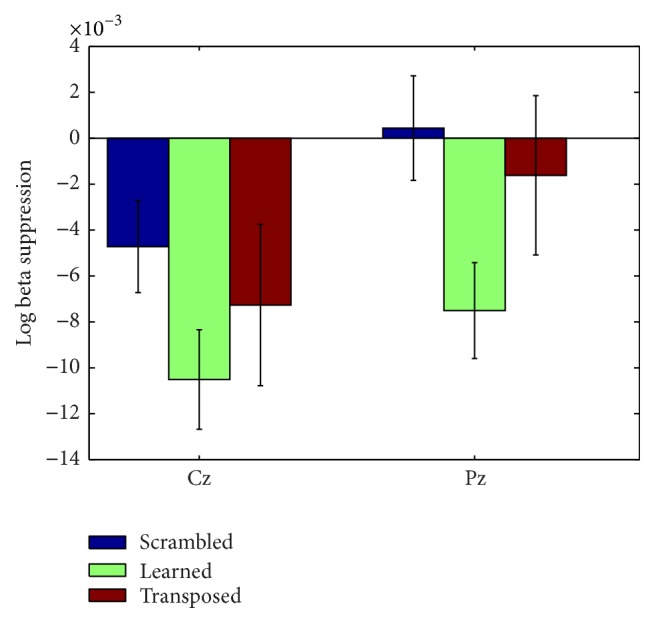
Beta suppression at electrodes Cz and Pz. Error bars represent standard error of the mean.

**Figure 6:**
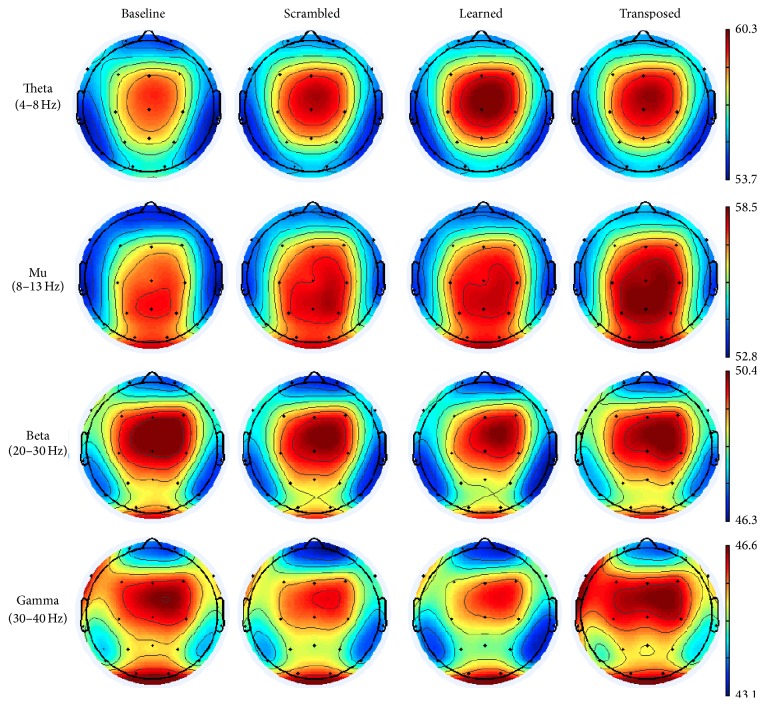
Scalp distribution of different frequency band activities. Units for power heat maps are microvolts squared.

Four additional repeated measure ANOVAs were calculated, one each for the theta, mu, beta, and gamma frequency bands, with electrode (19) and condition (3) factors (power tables of condition by electrode for each frequency band are available in Supplementary Materials). Within-subject effects revealed a main effect of electrode for the mu (*F*(18,270) = 1.7, *p* = 0.04) and theta bands (*F*(18,270) = 3.508, *p* = 1.0*e* − 3). No main effect was observed for condition; however the theta (*F*(36,540) = 1.59, *p* = 0.2), beta (*F*(36,540) = 1.59, *p* = 0.02), and gamma (*F*(36,540) = 1.656, *p* = 0.01) bands all revealed a significant interaction between electrodes and conditions. As seen in Figure 6, the activity across all frequency bands was centered over the midline electrodes. Examining these electrodes, both Cz and Pz revealed a trend in the predicted direction across conditions in the beta band, with learned melody exhibiting greatest suppression, followed next by transposed and lastly by the scrambled control melody. A significant main effect was observed for condition at these two recording sites (*F*(2,14) = 7.12, *p* = 0.007). All three musical conditions exhibited suppression with regard to the baseline at Cz, and at Pz the scrambled melody control showed a slight enhancement (Figure 5). Overall Cz showed greater suppression than Pz for all three conditions, though the difference between learned and scrambled melodies was larger at Pz. As the beta band has been shown to play a role in perception of sounds [29, 37] and the effect was only observed over sensorimotor cortex, this finding supports the hypothesis that the motor system may be involved in perception of musical sequences. No significant correlations were found between beta suppression at these sites and years of musical training, pitch-recognition-production difference scores, or length of time to reach error-free performance on the first day of training (statistics reported in Table 2). Lack of correlation between brain responses and these behavioral measures indicated that prior musical experience or aptitude is not likely influencing the neural physiological responses at the level of individuals. However, when musical experience was included as a between-subjects factor of group (musically experienced, musically naive) and repeated measures ANOVA with factors of central electrode (C3, Cz, C4, and Pz) and song condition (control, learned, and transposed), there was a main effect of electrode (*F*(3,12) = 10.84, *p* = 0.001) and an interaction between electrode by group (*F*(3,12) = 8.42, *P* = 0.003). The greatest group differences were at C3, with nonmusicians exhibiting greater beta suppression, and C4 where musically experienced participants exhibited greater beta suppression.

As cortical alpha power is variable in its peak frequency across the population [38] we performed an additional multivariate analysis on mu power that was integrated over a band defined by an individual's mu peak frequency within a fixed bandwidth (8–20 Hz, the frequency window between theta and beta cutoffs) [39]. Repeated measures ANOVA with factors of electrode (19) and condition (3) revealed no main effect of condition (*F*(2,30) = 0.43, *p* = 0.657) or an interaction between condition and electrode (*F*(36,540) = 0.68, *p* = 0.92). Including a between-subjects factor of group (2) in a repeated measure ANOVA with central electrode (C3, Cz, C4, and Pz) and condition (3) factors revealed a within-subjects interaction of electrode by musical experience (*F*(3,42) = 2.94, *p* = 0.044). The musician group showed virtually no suppression at electrodes C3 and Pz, relative to nonmusicians who exhibited relatively high levels of suppression. The interaction was not further explained by the addition of condition, as the three-way interaction was not significant (*F*(6,84) = 1.28, *p* = 0.27), and the differences between musicians and nonmusicians are greatest at the lateralized electrodes (C3 and C4). Power values are available in the Supplementary Materials for individual mu peak power spectra for all electrodes by condition and for central cluster of electrodes by group.

## 5. Discussion

The present study reports a novel finding that cortical audiomotor system activity, as evidenced by EEG, is sensitive to the effects of auditory sequence manipulations when the sounds are associated with movement. When subjects heard a melody they learned to play and a transposed version of that melody, they exhibited suppression of the beta band relative to baseline and relative to a scrambled melody control. The level of motor system engagement indexed by beta suppression was greater in response to listening to learned melodies than listening to the transposed version of these melodies. Suppression of the beta rhythm while listening to transposed versions of the learned melody indicates a role for motor system associations with the sequential aspects of an auditory stimulus. The present study builds on previous work by Lahav et al. [8] who reported motor system activity in the form of a blood oxygen level dependent (BOLD) signal when listening to the same learned melody. Lahav et al. further demonstrated partial motor system activation when subjects heard a novel melody composed of notes from the learned melody. They interpreted the partial activation as evidence that the motor system was sensitive to associations formed between single notes and single finger movements (recall one note per finger on the right hand). We hypothesized that the difference between motor system engagement levels for learned melodies and scrambled melodies of the same note set could be explained by an audiomotor system sensitivity to sequences of sounds. The evidence currently reported supports this hypothesis. A logical extension of the work would compare the levels of motor system engagement while listening to the transposed melody and scrambled melody (same notes and different sequence from learned song). If the motor system is sensitive to both single note (pitch) and sequence information, then the sum of motor activation between these two conditions should approximate the level observed when subjects listen to the melody they learned to play.

Suppression of beta rhythms is associated with motor engagement. Caetano et al. [29] reported that the beta frequency band desynchronizes in anticipation of actions, hearing, and seeing that same action, followed by rebound synchronization after completion of the event. They also reported that the mu rhythm followed a similar time course of suppression and rebound enhancement, however with slight delays in rebound compared to the beta rhythm. During the sound only condition, mu and beta responses exhibited less suppression during the anticipatory phase but rebound at the same latency as visual based stimuli. This suggests that prestimulus suppression is related to movement preparation or planning. Boonstra et al. [30] also report a similar beta suppression during auditory perception and a pretap suppression with rebound enhancement when subjects tapped along with a steady, rhythmic sound. Both beta and mu frequency bands are seen as the two most active bands in terms of phase coherence between a cerebral network engaged during rhythmic sound tap synchronizing [40]. Further evidence to support the relevance of beta band in musical sequencing or rhythmic processing comes from reports of activity centered around 25 Hz in response to missing (expected) rhythmic sounds [41–43] and from its proposed role in modulating perceived beat structure [37].

Rather than predicted mu suppression, mu enhancement was observed across all conditions. The trend was similar across electrodes in the sensorimotor scalp region. Scrambled and learned song conditions had similar low levels of enhancement, while the transposed song enhanced mu significantly greater than the other two conditions. Mu suppression was predicted based on past work identifying mu rhythms as having high power during rest and suppressed during visual, auditory, and audiovisual input sans movement [28]. However, the stimuli used by McGarry et al. [28] were not musical in nature, as the action and sounds were tearing a sheet of paper. Prediction of action sounds in the absence of visual input is difficult. Music and language, conforming to grammatical rules, can build predictions of future sounds based on the relationships between and sequences of past sounds. To assume that the same neural system predicts visual and auditory movement related stimuli may be an error, even though auditory information can facilitate visual processing. While the mu rhythm is sensitive to auditory information [28] and plays a role in visual to motor and audio transformations in terms of reading sheet music [33], it may not play a direct role in audiomotor processing by itself. Caetano et al. [29] report that the mu rhythm responded more robustly to tapping on a drum when there is somatosensory feedback. Pantomiming the same tapping action in the absence of a surface to tap on fails to suppress the mu rhythm in the same way as a tap with tactile feedback. Listening to a melodic sequence that has motor associations may not suppress mu rhythms as the experience does not include the sensation, or perhaps even simulation of a tactile response. However one should draw comparisons cautiously as previously reported mu responses to tapping indicate a response to a discrete movement, whereas in the present study neural responses were averaged over several discrete sound-action pairings.

Mu enhancement, greatest while listening to the transposed song, may reflect an inhibitory response, rather than motor system preparation simply associated with movement. The inhibition timing hypothesis [44, 45] interprets event related increases of mu power as an inhibition response during intensive activity in other cortical rhythms. For instance, mu may signify the inhibition of motor output, such that changes in beta rhythms sequencing motor commands remain a simulation, unable to affect actual muscles. The hypothesis further proposes a role of mu enhancement as a main source of synchronization in cortical rhythms to synchronize neuronal timing. Given the strong temporal structure of musical sounds, it may be reasonable to expect mu power to increase during perception of more complex sound passages that require additional sequencing resources. It is possible that listening to a transposition requires more cortical resources such that greater demands are placed on the timing mechanisms critical to sound sequencing. If mu enhancement reflects increased difficulty of auditory sequencing, then listening to complex rhythmic patterns may elicit greater enhancement of mu rhythms than simple rhythmic patterns.

We additionally report a novel inclusion of musically experienced subjects with the piano learning ear training task. At the individual subject level, previous music experience was not associated with any other measure, except the length of time it took to complete the first day of ear training. At the group level, previous musical training was associated with lower levels of mu and beta suppression over the left sensorimotor cortex. For instrumentalists who have experience associating right hand movements with pitch perception in music performance, this may represent a more efficient use of cortical resources. The right sensorimotor cortex, contralateral to piano trained hand, exhibited greater beta suppression in musically experienced subjects. This might be explained in part by previous reports indicating a preferential role of the right hemisphere in relative pitch processing [46]. Musical training may be represented in this case by increased recruitment of right hemisphere sensorimotor networks during pitch processing, resulting in increased suppression. Musical experience may explain effects in the present data, but caution is urged in generalization of these findings, as we did not specifically recruit musicians, and only two of our subjects were regularly playing music at the time of their participation.

Subjects in the present study learned to play the melodic sequence by ear, as evidenced by the changes in length of time to complete training across sessions, replicating past use of this behavioral task [17]. The time to complete training followed an exponential decay curve, also showing a collapse of variance across subjects. Our novel inclusion of subjects with previous musical experience had an effect on the length of time to learn the song on the first day of training, but after five days of ear training and piano playing, differences between subjects groups were negligible. The two population groups, both piano naive, did not have significantly different P-R-P test scores before or after training. As previous reports from Lahav et al. [17] were performed with only musically naive subjects, the present work extends this task as a viable training with little differences for both the musically naive and musically experienced but piano naive subjects.

Taken in light of previous findings, enhanced mu and suppressed beta might indicate greater cortical demands in response to sounds associated with a motor action. As previous authors [8] hypothesized a trained association between discrete musical pitches and discrete finger movements, an extension of this work could make use of the temporal resolution of EEG and design the posttraining assessment to focus on reactions to discrete musical notes or a sequence of multiple notes to test whether cortical oscillations are recruited according to the time intervals related to sequence complexity. Relevant work from functional imaging suggests increases in auditory and premotor cortical activity proportional to difficulty of tapped rhythms [10]. If the motor system contributes to offline processing of sequential or rhythmic sounds at the level of discrete sounds, then one could predict beta desynchronization or mu synchronization in response to heard sounds without movement.

## Supplementary Material

The supplementary materials are provided to share additional comparisons of condition effects at different frequency bands and calculated at electrode sites than those discussed in the main text. These data compliment the topographic maps represented in figure 6. We also include statistical reports for group comparison effects that were not elaborated in detail in the main text. These represent output from SPSS for repeated measures ANOVAs, described in the methods section.

## Figures and Tables

**Table 1:** Self-reported demographics for musical experience in subjects with past musical exposure.

Age	Gender	Instrument(s)	Years of training	Currently playing	Hours per week
18	M	Saxophone	4	No	0
19	F	Guitar, flute	1	No	0
19	M	Guitar, vocals	4	Yes	1.5
20	F	Voice, viola	5	No	0
20	F	Flute	2	No	0
20	F	Clarinet, saxophone	7.5	No	0
20	M	Violin	2	No	0
20	M	Drum set, tabla	6	Yes	2

**Table 2:** Nonparametric correlations between behavioral measures and beta band activity.

Beta band behavioral correlations												
	C3 control	C3 learned	C3 transposed	CZ control	CZ learned	CZ transposed	C4 control	C4 learned	C4 transposed	PZ control	PZ learned	PZ transposed
Spearman's rho												
Completion time day 1	.833	.914	.935	.974	.284	.766	.948	.427	.300	.884	.152	.791
	−.057	.029	−.022	.009	.286	.081	−.018	.213	.277	−.040	.375	.072
	16	16	16	16	16	16	16	16	16	16	16	16
Years of musical training	.529	.912	.899	.419	.412	.335	.325	.296	.091	.958	.072	.402
	.170	.030	−.035	−.217	−.220	−.258	−.263	−.279	−.436	−.014	−.461	−.225
	16	16	16	16	16	16	16	16	16	16	16	16
Pre- and post difference PRP test	.491	.184	.158	.401	.578	.349	.014	.410	.559	.320	.704	.644
	−.186	−.350	−.370	−.226	−.150	−.251	−.599	−.221	−.158	−.265	−.103	−.125
	16	16	16	16	16	16	16	16	16	16	16	16
