# Supplementary material for: Musical Sequence Learning and EEG Correlates of Audiomotor Processing
Source: Behav Neurol. 2015 Oct 7;2015:638202. doi: 10.1155/2015/638202 (PMC4617417; doi:10.1155/2015/638202)
Supplement: Supplementary file 1 — The supplementary materials are provided to share additional comparisons of condition effects at different frequency bands and calculated at electrode sites than those discussed in the main text. These data compliment the topographic maps represented in figure 6. We also include statistical reports for group comparison effects that were not elaborated in detail in the main text. These represent output from SPSS for repeated measures ANOVAs, described in the methods section. [file 638202.f1.pdf]

frequency	electrode	condition	Mean	Std. Error	95% Confidence Interval	
					Lower Bound	Upper Bound
Mu	C3	Scrambled	.009	.006	-.004	.022
		Learned	.014	.004	.005	.022
		Transposed	.020	.005	.009	.031
	C4	Scrambled	.013	.005	.001	.024
		Learned	.011	.004	.003	.019
		Transposed	.017	.005	.007	.027
Beta	C3	Scrambled	-.005	.002	-.009	.000
		Learned	-.011	.003	-.017	-.004
		Transposed	-.001	.007	-.015	.014
	C4	Scrambled	-.005	.003	-.011	.001
		Learned	-.007	.003	-.014	-6.626E-005
		Transposed	.002	.006	-.010	.014
Theta	C3	Scrambled	.764	.008	.747	.781
		Learned	.758	.008	.741	.775
		Transposed	.768	.010	.747	.789
	C4	Scrambled	.765	.008	.748	.782
		Learned	.762	.008	.745	.780
		Transposed	.772	.008	.754	.790
Gamma	C3	Scrambled	-.004	.004	-.013	.005
		Learned	-.010	.005	-.020	-1.003E-006
		Transposed	.010	.013	-.017	.037
	C4	Scrambled	-.010	.005	-.021	.001
		Learned	-.014	.006	-.028	.000
		Transposed	.004	.011	-.019	.027

Figure 9. Examining the effect of Frequency Band by Conditions at electrodes C3 and C4

SUPPLEMENTAL DATA

Figure 10. Examining the effect of Electrode by Condition in the Beta frequency band

electrode	condition	Mean	Std. Error	95% Confidence Interval Lower Bound	95% Confidence Interval Upper Bound
F7	Scrambled	-.013	.004	-.021	-.004
	Learned	-.010	.005	-.021	.001
	Transposed	.001	.008	-.016	.017
F8	Scrambled	-.011	.004	-.019	-.002
	Learned	-.008	.005	-.017	.002
	Transposed	.000	.007	-.015	.015
F3	Scrambled	-.010	.004	-.019	-.001
	Learned	-.016	.007	-.030	-.002
	Transposed	-.002	.006	-.015	.010
Fz	Scrambled	-.007	.003	-.013	-.001
	Learned	-.008	.003	-.013	-.003
	Transposed	-.005	.004	-.013	.004
F4	Scrambled	-.007	.003	-.014	.001
	Learned	-.004	.004	-.012	.004
	Transposed	-.002	.006	-.014	.010
C3	Scrambled	-.005	.002	-.009	.000
	Learned	-.011	.003	-.017	-.004
	Transposed	-.001	.007	-.015	.014
Cz	Scrambled	-.005	.002	-.009	.000
	Learned	-.011	.002	-.015	-.006
	Transposed	-.007	.004	-.015	.000
C4	Scrambled	-.005	.003	-.011	.001
	Learned	-.007	.003	-.014	-6.626E-005
	Transposed	.002	.006	-.010	.014
P3	Scrambled	.000	.002	-.004	.004
	Learned	-.007	.002	-.012	-.002
	Transposed	9.254E-005	.005	-.010	.010
Pz	Scrambled	.000	.002	-.004	.005
	Learned	-.008	.002	-.012	-.003
	Transposed	-.002	.003	-.009	.006
P4	Scrambled	-.001	.003	-.007	.004
	Learned	-.004	.003	-.010	.001
	Transposed	.002	.004	-.005	.010
T5	Scrambled	-.002	.002	-.007	.002
	Learned	-.007	.003	-.013	.000
	Transposed	.005	.011	-.017	.028
T6	Scrambled	-.004	.003	-.011	.003
	Learned	-.008	.003	-.015	-.001
	Transposed	.005	.005	-.006	.016
O1	Scrambled	-.005	.002	-.010	.001
	Learned	-.005	.002	-.009	-.001
	Transposed	-.003	.003	-.010	.003
O2	Scrambled	-.005	.002	-.010	.000
	Learned	-.005	.002	-.008	-.001
	Transposed	-.002	.002	-.007	.003
T3	Scrambled	-.008	.003	-.013	-.002
	Learned	-.012	.007	-.028	.004
	Transposed	.003	.009	-.016	.023
T4	Scrambled	-.010	.004	-.019	-.001
	Learned	-.014	.007	-.029	.001
	Transposed	.000	.006	-.012	.013
FP1	Scrambled	-.009	.004	-.017	-.001
	Learned	-.005	.004	-.013	.003
	Transposed	-.003	.005	-.013	.007
FP2	Scrambled	-.009	.004	-.017	-.001
	Learned	-.006	.003	-.013	.001
	Transposed	-.002	.005	-.012	.009

Figure 11. Examining the effect of Electrode by Condition in the Gamma frequency band.

electrode	condition	Mean	Std. Error	95% Confidence Interval Lower Bound	95% Confidence Interval Upper Bound
F7	Scrambled	-.011	.007	-.026	.004
	Learned	-.011	.010	-.033	.010
	Transposed	.010	.014	-.019	.039
F8	Scrambled	-.016	.008	-.032	.000
	Learned	-.011	.010	-.033	.011
	Transposed	-.002	.014	-.032	.027
F3	Scrambled	-.011	.008	-.028	.005
	Learned	-.016	.011	-.040	.008
	Transposed	.004	.013	-.023	.031
Fz	Scrambled	-.010	.006	-.023	.002
	Learned	-.009	.007	-.025	.006
	Transposed	.001	.010	-.020	.022
F4	Scrambled	-.009	.007	-.024	.005
	Learned	-.006	.008	-.023	.012
	Transposed	.001	.012	-.024	.026
C3	Scrambled	-.004	.004	-.013	.005
	Learned	-.010	.005	-.020	-1.003E-006
	Transposed	.010	.013	-.017	.037
Cz	Scrambled	-.009	.004	-.017	.000
	Learned	-.011	.003	-.018	-.004
	Transposed	-.001	.008	-.017	.015
C4	Scrambled	-.010	.005	-.021	.001
	Learned	-.014	.006	-.028	.000
	Transposed	.004	.011	-.019	.027
P3	Scrambled	-.004	.003	-.010	.002
	Learned	-.011	.003	-.018	-.004
	Transposed	.002	.009	-.017	.022
Pz	Scrambled	-.006	.003	-.013	.001
	Learned	-.012	.003	-.019	-.006
	Transposed	-.001	.007	-.016	.013
P4	Scrambled	-.007	.004	-.016	.002
	Learned	-.015	.004	-.024	-.006
	Transposed	.001	.008	-.015	.017
T5	Scrambled	-.008	.004	-.017	.002
	Learned	-.014	.004	-.022	-.006
	Transposed	.004	.014	-.026	.034
T6	Scrambled	-.009	.006	-.021	.003
	Learned	-.017	.006	-.030	-.004
	Transposed	.002	.010	-.020	.024
O1	Scrambled	-.009	.003	-.017	-.002
	Learned	-.011	.004	-.019	-.003
	Transposed	-.008	.005	-.019	.003
O2	Scrambled	-.009	.004	-.017	-.001
	Learned	-.008	.003	-.015	-.001
	Transposed	-.006	.005	-.017	.005
T3	Scrambled	-.006	.005	-.017	.006
	Learned	-.018	.011	-.041	.005
	Transposed	.017	.013	-.012	.045
T4	Scrambled	-.014	.008	-.031	.003
	Learned	-.022	.010	-.044	.001
	Transposed	.001	.013	-.027	.029
FP1	Scrambled	-.011	.007	-.026	.004
	Learned	-.006	.009	-.025	.013
	Transposed	.001	.010	-.019	.022
FP2	Scrambled	-.014	.006	-.028	-.001
	Learned	-.009	.007	-.025	.007
	Transposed	.002	.011	-.022	.026

Figure 12. Examining the effect of Electrode by Condition in the Mu frequency band.

electrode	condition	Mean	Std. Error	95% Confidence Interval Lower Bound	95% Confidence Interval Upper Bound
F7	Scrambled	.005	.004	-.004	.013
	Learned	.004	.003	-.001	.010
	Transposed	.009	.005	-.003	.020
F8	Scrambled	.008	.004	.000	.017
	Learned	.007	.004	-.002	.017
	Transposed	.013	.005	.002	.023
F3	Scrambled	.011	.005	.001	.021
	Learned	.012	.004	.004	.020
	Transposed	.018	.005	.007	.029
Fz	Scrambled	.011	.004	.003	.020
	Learned	.010	.003	.003	.016
	Transposed	.017	.005	.006	.028
F4	Scrambled	.012	.004	.003	.020
	Learned	.011	.004	.004	.019
	Transposed	.017	.005	.007	.028
C3	Scrambled	.009	.006	-.004	.022
	Learned	.014	.004	.005	.022
	Transposed	.020	.005	.009	.031
Cz	Scrambled	.006	.005	-.005	.017
	Learned	.008	.004	.000	.016
	Transposed	.017	.006	.005	.029
C4	Scrambled	.013	.005	.001	.024
	Learned	.011	.004	.003	.019
	Transposed	.017	.005	.007	.027
P3	Scrambled	.007	.006	-.006	.020
	Learned	.007	.006	-.005	.019
	Transposed	.016	.005	.004	.028
Pz	Scrambled	.005	.006	-.008	.017
	Learned	.005	.005	-.006	.016
	Transposed	.014	.006	.001	.026
P4	Scrambled	.012	.006	-.001	.025
	Learned	.007	.005	-.003	.017
	Transposed	.013	.004	.004	.021
T5	Scrambled	.009	.006	-.003	.021
	Learned	.007	.005	-.004	.019
	Transposed	.016	.005	.006	.026
T6	Scrambled	.011	.005	.000	.023
	Learned	.008	.005	-.003	.018
	Transposed	.012	.004	.004	.020
O1	Scrambled	.003	.004	-.006	.012
	Learned	.005	.004	-.004	.013
	Transposed	.010	.005	.000	.021
O2	Scrambled	.002	.004	-.006	.010
	Learned	.006	.004	-.002	.013
	Transposed	.012	.004	.003	.021
T3	Scrambled	.007	.006	-.005	.018
	Learned	.010	.005	.001	.020
	Transposed	.013	.005	.002	.024
T4	Scrambled	.013	.006	.000	.026
	Learned	.009	.005	-.002	.020
	Transposed	.013	.005	.002	.025
FP1	Scrambled	.012	.004	.004	.019
	Learned	.010	.003	.002	.017
	Transposed	.018	.006	.006	.030
FP2	Scrambled	.012	.003	.005	.019
	Learned	.011	.004	.004	.019
	Transposed	.020	.005	.009	.031

Figure 13. Examining the effect of Electrode by Condition in the Theta frequency band.

electrode	condition	Mean	Std. Error	95% Confidence Interval Lower Bound	95% Confidence Interval Upper Bound
F7	Scrambled	.764	.006	.751	.777
	Learned	.766	.006	.753	.779
	Transposed	.777	.009	.758	.797
F8	Scrambled	.760	.007	.745	.775
	Learned	.764	.006	.751	.776
	Transposed	.771	.010	.751	.791
F3	Scrambled	.759	.007	.743	.774
	Learned	.753	.006	.740	.766
	Transposed	.767	.009	.748	.785
Fz	Scrambled	.754	.007	.740	.768
	Learned	.753	.007	.739	.767
	Transposed	.756	.007	.742	.771
F4	Scrambled	.763	.007	.747	.779
	Learned	.766	.007	.750	.781
	Transposed	.767	.007	.752	.783
C3	Scrambled	.764	.008	.747	.781
	Learned	.758	.008	.741	.775
	Transposed	.768	.010	.747	.789
Cz	Scrambled	.755	.009	.736	.774
	Learned	.749	.009	.731	.768
	Transposed	.752	.009	.733	.772
C4	Scrambled	.765	.008	.748	.782
	Learned	.762	.008	.745	.780
	Transposed	.772	.008	.754	.790
P3	Scrambled	.762	.009	.743	.781
	Learned	.755	.010	.734	.775
	Transposed	.762	.009	.742	.781
Pz	Scrambled	.751	.009	.732	.770
	Learned	.743	.010	.723	.763
	Transposed	.749	.009	.730	.768
P4	Scrambled	.760	.008	.742	.778
	Learned	.757	.009	.738	.775
	Transposed	.763	.008	.746	.780
T5	Scrambled	.774	.009	.755	.794
	Learned	.770	.009	.750	.789
	Transposed	.782	.011	.758	.806
T6	Scrambled	.769	.007	.754	.783
	Learned	.764	.007	.750	.779
	Transposed	.777	.008	.760	.794
O1	Scrambled	.777	.011	.754	.800
	Learned	.777	.011	.754	.799
	Transposed	.779	.011	.755	.802
O2	Scrambled	.776	.009	.757	.795
	Learned	.777	.009	.759	.795
	Transposed	.779	.009	.761	.798
T3	Scrambled	.780	.009	.761	.800
	Learned	.776	.009	.757	.795
	Transposed	.791	.009	.771	.811
T4	Scrambled	.772	.009	.753	.791
	Learned	.769	.009	.748	.789
	Transposed	.783	.010	.763	.803
FP1	Scrambled	.758	.006	.746	.770
	Learned	.762	.006	.749	.774
	Transposed	.764	.007	.748	.779
FP2	Scrambled	.757	.007	.742	.772
	Learned	.760	.006	.746	.773
	Transposed	.764	.008	.748	.780

Figure 14. Mu suppression values for individually identified mu peak frequencies.

MusicalTraining * Electrode * Condition

MusicalTraining	Electrode	Condition	Mean	Std. Error	95% Confidence Interval Lower Bound	Upper Bound
no	C3	control	-.012	.007	-.028	.004
		learned	-.008	.008	-.024	.009
		transpose	-.013	.008	-.030	.003
	CZ	control	-.010	.007	-.024	.004
		learned	.000	.007	-.016	.015
		transpose	-.006	.007	-.022	.010
	C4	control	-.012	.006	-.026	.001
		learned	-.001	.008	-.018	.017
		transpose	.003	.007	-.013	.018
	PZ	control	-.007	.006	-.021	.006
		learned	-.003	.006	-.016	.010
		transpose	-.010	.006	-.024	.004
yes	C3	control	.006	.007	-.010	.022
		learned	.001	.008	-.016	.017
		transpose	-.003	.008	-.019	.014
	CZ	control	.002	.007	-.012	.016
		learned	.001	.007	-.015	.016
		transpose	-.003	.007	-.019	.012
	C4	control	.001	.006	-.012	.014
		learned	-.006	.008	-.023	.011
		transpose	-.008	.007	-.024	.007
	PZ	control	.004	.006	-.009	.017
		learned	-.003	.006	-.016	.010
		transpose	-.004	.006	-.017	.010

Figure 15. Beta suppression values for group differences over central electrodes.

MusicalTraining * electrode * condition

MusicalTraining	electrode	condition	Mean	Std. Error	95% Confidence Interval Lower Bound	Upper Bound
no	C3	control	-.007	.003	-.014	.000
		learned	-.012	.004	-.021	-.002
		transposed	-.006	.010	-.027	.014
	CZ	control	-.003	.003	-.010	.003
		learned	-.009	.003	-.016	-.002
		transposed	-.007	.005	-.018	.004
	C4	control	-.003	.004	-.012	.006
		learned	-.003	.005	-.013	.007
		transposed	.002	.008	-.016	.020
	PZ	control	.001	.003	-.006	.008
		learned	-.004	.003	-.011	.002
		transposed	-.001	.005	-.012	.010
yes	C3	control	-.002	.003	-.009	.005
		learned	-.009	.004	-.019	-4.223E-006
		transposed	.005	.010	-.016	.026
	CZ	control	-.006	.003	-.012	.000
		learned	-.012	.003	-.019	-.005
		transposed	-.008	.005	-.019	.003
	C4	control	-.007	.004	-.016	.002
		learned	-.012	.005	-.022	-.002
		transposed	.002	.008	-.016	.020
	PZ	control	.000	.003	-.007	.007
		learned	-.011	.003	-.017	-.005
		transposed	-.002	.005	-.013	.009

Figure 16. Mu suppression values for individually identified mu peak frequencies, showing gorup differences over central electrodes.

MusicalTraining * Electrode * Condition

MusicalTraining	Electrode	Condition	Mean	Std. Error	95% Confidence Interval Lower Bound	95% Confidence Interval Upper Bound
no	C3	control	-.012	.007	-.028	.004
		learned	-.008	.008	-.024	.009
		transpose	-.013	.008	-.030	.003
	CZ	control	-.010	.007	-.024	.004
		learned	.000	.007	-.016	.015
		transpose	-.006	.007	-.022	.010
	C4	control	-.012	.006	-.026	.001
		learned	-.001	.008	-.018	.017
		transpose	.003	.007	-.013	.018
	PZ	control	-.007	.006	-.021	.006
		learned	-.003	.006	-.016	.010
		transpose	-.010	.006	-.024	.004
yes	C3	control	.006	.007	-.010	.022
		learned	.001	.008	-.016	.017
		transpose	-.003	.008	-.019	.014
	CZ	control	.002	.007	-.012	.016
		learned	.001	.007	-.015	.016
		transpose	-.003	.007	-.019	.012
	C4	control	.001	.006	-.012	.014
		learned	-.006	.008	-.023	.011
		transpose	-.008	.007	-.024	.007
	PZ	control	.004	.006	-.009	.017
		learned	-.003	.006	-.016	.010
		transpose	-.004	.006	-.017	.010
